# Clinical decision support improves quality of telephone triage documentation - an analysis of triage documentation before and after computerized clinical decision support

**DOI:** 10.1186/1472-6947-14-20

**Published:** 2014-03-20

**Authors:** Frederick North, Debra D Richards, Kimberly A Bremseth, Mary R Lee, Debra L Cox, Prathibha Varkey, Robert J Stroebel

**Affiliations:** 1 Primary Care Internal Medicine, Mayo Clinic, Rochester, Minnesota, USA; 2 Department of Nursing, Employee and Community Health, Mayo Clinic, Rochester, Minnesota, USA; 3 Department of Nursing Administration, Mayo Clinic, Rochester, Minnesota, USA; 4 Division of Preventive, Occupational and Aerospace Medicine, Mayo Clinic, Rochester, Minnesota, USA

**Keywords:** Telephone triage, Clinical decision support, Triage documentation

## Abstract

**Background:**

Clinical decision support (CDS) has been shown to be effective in improving medical safety and quality but there is little information on how telephone triage benefits from CDS. The aim of our study was to compare triage documentation quality associated with the use of a clinical decision support tool, ExpertRN^©^.

**Methods:**

We examined 50 triage documents before and after a CDS tool was used in nursing triage. To control for the effects of CDS training we had an additional control group of triage documents created by nurses who were trained in the CDS tool, but who did not use it in selected notes. The CDS intervention cohort of triage notes was compared to both the pre-CDS notes and the CDS trained (but not using CDS) cohort. Cohorts were compared using the documentation standards of the American Academy of Ambulatory Care Nursing (AAACN). We also compared triage note content (documentation of associated positive and negative features relating to the symptoms, self-care instructions, and warning signs to watch for), and documentation defects pertinent to triage safety.

**Results:**

Three of five AAACN documentation standards were significantly improved with CDS. There was a mean of 36.7 symptom features documented in triage notes for the CDS group but only 10.7 symptom features in the pre-CDS cohort (p < 0.0001) and 10.2 for the cohort that was CDS-trained but not using CDS (p < 0.0001). The difference between the mean of 10.2 symptom features documented in the pre-CDS and the mean of 10.7 symptom features documented in the CDS-trained but not using was not statistically significant (p = 0.68).

**Conclusions:**

CDS significantly improves triage note documentation quality. CDS-aided triage notes had significantly more information about symptoms, warning signs and self-care. The changes in triage documentation appeared to be the result of the CDS alone and not due to any CDS training that came with the CDS intervention. Although this study shows that CDS can improve documentation, further study is needed to determine if it results in improved care.

## Background

Clinical decision support (CDS) has been shown to be effective in improving medical safety and quality in a number of ways. Much of the current literature has demonstrated the benefits of CDS in medication prescribing, preventive services, and prophylaxis [1]. There has been little published on the potential impact of CDS on a critical nursing function, telephone triage.

Telephone calls requiring medical advice make up a significant proportion of the total calls to a medical practice. For example, a large group practice in the US which handles about 1 million telephone calls per month found that 16% of their calls required medical advice [2]. These calls are frequently about a new symptom and the caller wants medical advice on what to do. In the US, medical practices vary considerably in how these calls are handled; there is no required uniform standard.

For both the patient and the practice there are risks associated with these calls. For the patient, there can be wide variability in the expertise of the individual answering the telephone, especially between calls handled during office hours and those after-hours. Some practices, for example, leave their after-hours calls in the hands of answering service operators who make medical decisions without the direct help of a nurse or physician. Hildebrandt, in a study from the US (Colorado) found that 56% of the family medicine practices and 65% of the internal medicine practices used an answering service for after-hours calls [3]. He found that the answering services had no nurses to help with triage and the decision to go to an emergency department or call a doctor was put back in the caller’s hands. Pediatric practices in Colorado used an entirely different approach for their after-hours calls. Up to 84% of the pediatric practices used an after-hours call center which employed specifically trained triage nurses and used computer algorithms to help guide the nurses’ decisions [4]. Internationally, several countries have less variability in their after-hours telephone coverage than the US because they have nationwide systems for answering calls [5]. Foremost among the countries with standardized after-hours call coverage is the UK, which has National Health Service Direct, a call center with nurses using standardized algorithms to handle over 4 million calls a year [6].

Best processes for answering symptom calls during regular office hours have not been definitively identified. There is also no uniform standard for telephone triage performed during office hours. However, Poole in the US has made several suggestions for how pediatric calls should be handled to increase efficiency and decrease risk [7,8].

It is important for those doing telephone triage (triagists) to ask a minimum set of questions to hone in on critical factors that may indicate higher risk. When medical experts agree on a particular set of questions required for a specific symptom, the resulting question set can be used as a standard. For example, a set of questions to assess dehydration would be important to determine the severity of illness in a caller with diarrhea or vomiting. Answers to questions about urine output and lightheadedness help determine how aggressively to treat those with vomiting and diarrhea. The symptoms or signs associated with higher risk are called critical symptom indicators and they are essential for appropriate triage decision making. The resultant quality measure involving critical symptom indicators is a count of critical symptom indicator questions asked by the triagist compared to the number in the ideal set. Some studies using this measure of triage quality show that triage information gathering was rated as poor in 19% of calls [9]. In a study from the Netherlands using standardized callers and not using clinical decision support, an entire set of obligatory questions was asked in only 21% of the triage calls [10]. Even physicians taking calls were only able to average 7 out of 9 critical questions for diarrhea as a symptom [11]. It should be noted that these studies took place without the benefit of computerized clinical decision support. It stands to reason that a computer prompting a triagist with these questions could improve those results. Quality of triage documentation does not necessarily reflect the quality of triage. For example, a triage nurse could ask an entire set of critical questions to determine the severity of dehydration and then not document it. In this study we solely examine the changes in the quality of triage documentation before and after the implementation of a computerized clinical decision support tool for telephone triage.

## Methods

### Study design and overview

This was a retrospective study of telephone triage documents in electronic medical records. Our study design included one CDS intervention cohort and two control cohorts. The cohorts were: (1) triage documents from nurses before computerized CDS was available, (2) triage documents after CDS from the same nurses and, (3) triage documents from nurses during the same time as cohort 2 who were trained in CDS but had notes generated without CDS. The CDS intervention group was compared to the pre-intervention group (nurses were the same in both pre and post CDS intervention groups). The CDS group was also compared to a concurrent group (different nurses but same training). Because telephone triage with computerized CDS is a partnership of software to triagist [12], we used 2 controls. The triagist using the software has to have extra training with the software so it is possible that the extra training alone (no CDS) could improve triage documentation. To address the possible confounding factor of additional training we had a cohort of triage notes authored by nurses with additional training involved in CDS but who did not use CDS for those notes.

This study was approved by the Mayo Clinic Institutional Review Board.

### Practice setting

The study took place in the primary care practice of Mayo Clinic Rochester, Minnesota. The primary care practice has 141,543 patients. Patients under age 18 account for 15% of the primary care practice and those aged 65 or over are 16% of the total. Telephone calls about symptoms are transferred to nurses specifically trained in telephone triage.

### Clinical decision support tool

The clinical decision support software was ExpertRN^©^, a proprietary triage program of the Mayo Clinic. ExpertRN contains 140 symptom-related algorithms and was used for decision support in 56,421 primary care calls in 2010 and 65,705 calls in 2011. Each algorithm generally assesses a single symptom so that there are separate algorithms for chest pain, abdominal pain, headache, diarrhea, etc. At the start of the call, the triage nurse chooses the algorithm associated with the most urgent symptom if more than one symptom is present. Forty-three of the algorithms use extensive branching logic and each algorithm may contain as many as several hundred questions. Once a symptom algorithm is chosen, the software presents the triage nurse with groups of questions to ask the caller. Based on the responses to the initial questions, the software presents additional sets of questions which eventually branch to a disposition decision supported by the previous answers.

Symptom calls to the Mayo Clinic primary care practice are answered by nurses specifically trained in telephone triage and in the use of ExpertRN software. In most algorithms there are critical symptom indicator questions at the start of the structured dialog that will rapidly lead to an emergency recommendation or very urgent disposition. For example, callers describing chest pain associated with nausea, sweating, a crushing sensation or shortness of breath will be instructed to call 911 and to chew an aspirin if available. If the critical symptom indicators are not present then the algorithm proceeds to prompt questions until the software determines that there is enough information to make a disposition recommendation to the triage nurse. The disposition recommendation consists of two basic components: an urgency component (how soon care should be sought) and a place of care component (ambulance, emergency department, doctor’s office appointment, call to office, or self-care only). Care points (self-care recommendations) also may be served up by the software and tailored to symptoms and dispositions. These care points are software generated and often offer the nurse a wide range of possible recommendations for patient self-care after the assessment is done. The triage nurse can select from a menu of these care points and recommend the ones deemed appropriate by nursing judgment. It should be noted that the care points are not just for a home care disposition. For severe chest pain, the software delivers a care point of “chew and swallow one regular-strength aspirin (325 milligrams) or four low-dose aspirin (81 milligrams) as soon as possible…”. The care points the nurse selects are automatically documented in the triage call record by the software.

After ExpertRN generates the disposition, the triage nurse has the option of overriding the software recommendation. Callers are also asked whether they are in agreement with the recommendation, allowing the nurse further persuasive efforts or negotiation if needed. At the end of the call there is a seamless transfer of information from ExpertRN into the medical record. The triage note is automatically generated to conform to a standard Mayo Clinic triage note format. The software-generated triage note contains all the call registration data, the complete set of answers to the triage questions, the most urgent disposition, care points selected, and caller disagreement information. Nurses have free text fields to add information to the note and the entire medical note is open for them to edit. The mechanics of presenting appropriate and complete question sets in a logical sequence, recording answers, organizing the note, and transcribing the details into the document are all handled by the software. As a consequence, ExpertRN has extensive documentation support as well as clinical decision support. It should be noted that the CDS generated document can be greatly influenced by the choice of the algorithm and path, which is dependent on the critical thinking and skill of the triagist.

### Selection of triage documents to review

We selected triage notes in the medical record before April 2008 when there was no CDS and after April 2010 when CDS (ExpertRN) was in use and well established. There were 120 nurses with training in ExpertRN as of 2009. Of those 120 we identified 37 who had triage notes prior to April 2008 (before implementation of CDS) and after April 2010 when CDS had already been implemented for over a year. This group of nurses had created triage notes before and after the implementation of CDS, and so were their own controls after implementation of CDS. Of the 37, we randomly selected 25 triage nurses who authored the notes we studied. For each of the 25 nurses, we picked their last 2 triage notes before April 2008 (pre-CDS) and their first 2 triage notes starting May 1, 2010 (post-CDS). This gave us our pre-CDS cohort of 50 triage notes and our cohort of 50 triage notes using CDS (our post-CDS cohort), with both note cohorts authored by the same triage nurses.

ExpertRN requires additional training to master and so nurses trained in ExpertRN might produce substantially improved triage notes even if they did not actually use the decision support. To control for a change in triage note quality due to additional training, we used a concurrent control of 50 triage notes from nurses who completed standardized ExpertRN training but had not used ExpertRN for the cohort of notes we evaluated. These notes from nurses who had training in ExpertRN but fewer than 300 symptom assessments using ExpertRN in 2010 served as our control. We randomly selected 25 of the 36 triage nurses who fit the criteria and 22 of those 25 nurses were distinct from the pre-post group. These nurses still were using ExpertRN, and as a group had authored a mean of 149 notes (median 164, SD 86) using ExpertRN in 2010. The documents selected for review were the first two triage notes after May 1, 2010 for each of the 25 nurses (notes without use of CDS). This gave us an additional 50 triage note cohort from nurses trained in ExpertRN but with notes authored without ExpertRN. This note cohort served as a control for the effect of ExpertRN training. Nurses responsible for the triage notes were compared by level of education (bachelor’s degree or not) and length of work experience at Mayo Clinic. We were unable to compare total nursing work years, which would have required more personal inquiries about time away from nursing.

### Measures

We reviewed the triage document content using several different measures. For a measure of how content agreed with nursing standards, we used the American Academy of Ambulatory Care Nursing (AAACN) telephone triage documentation recommendations for our comparison [13]. The telephone triage documentation recommendations of the AAACN are based on 6 dimensions of triage information documentation. These dimensions are encounter characteristics, patient characteristics, contact characteristics, reason for the call, nursing actions and post-triage disposition action. In turn, each of these dimensions is made up of 3 to 6 specific criteria. The AAACN criteria were scored dichotomously as achieving the criteria or not.

We measured additional content by counting the total symptoms and signs contained in each document as well as the total care points and warning signs. We defined care points as separate instructions from the nurse about self-care. These are triage nurse recommendations to improve symptoms. Examples would be “take acetaminophen for pain” and “apply heating pad to the affected area” (these two examples would total 2 care points). We defined warning symptoms as symptom “red flags” to watch for that would necessitate change in care if they occurred. A generic warning to call back if symptoms changed was not sufficient to be counted as a warning symptom. To be counted, warning signs had to be specific signs or symptoms to watch for.

We also assessed the overall structure of the note and tallied note organization defects defined as content in the wrong note section. Our triage notes are structured in the electronic medical record so that features of the history are contained in the “History” section of the note, the disposition is contained in the “Impression/Plan” section of the note, and other call demographics are in the header. When reading notes or for software analysis of records, it is important that the information is put in the correct note section. In reviewing the notes, we examined how the information in the notes was organized and if data elements were out of place we classified them as organizational defects. There were 3 sections in each note where data could be misplaced so there was a total of 150 possible organizational defects in the sample of 50.

Triage notes were assessed for the presence of critical symptom indicators using established lists in the literature [14]. Triage urgency was considered specific if a definite time frame for a disposition was in the triage note. For example, triage urgency was considered specific if the triage note indicated an office visit was needed and stipulated a specific time frame of hours or days when the visit should take place.

Occasionally, triagists will give more than one specific recommendation which can confuse the caller. We reviewed the documents for instances of more than one disposition.

Nurse reviewers also looked for major documentation defects that could indicate a quality problem. They looked for triage documents that could stand on their own to indicate that the triage quality was adequate. Our standard was that the triage documentation should not require additional audio review of the triage call to ascertain patient safety. Triage notes with major documentation defects were ones that required additional audio reviews to ascertain patient safety. Nurse reviewers indicated the reason for a major documentation defect in a free text field.

### Documentation review process

Working together as a group on a test sample of 16 records, three nurses made additions and modifications to the AAACN criteria to produce the criteria that they could agree upon independently (Table 1). The 16 record test sample also gave us preliminary information needed to determine that the final sample size of 50 records would be adequate to show significant changes in our major measures. There were no a priori sample size calculations before the test sample. Using a REDCap database [15] to collect abstraction data, the same three nurses independently reviewed all 3 cohorts of 50 triage records each. In addition to the AAACN criteria, for each triage record the nurses also counted the entire documented positive and negative associated signs and symptoms related to symptom triage, total care points, and total warning symptoms given.

**Table 1:** **Triage note groups and specific documentation criteria used (**^+^is CDS generated; *is CDS prompted)

**Triage documentation category**	**Triage note documentation elements (present/absent)**
Encounter characteristics	^+^1. Date/Time of encounter
	*2. Telephone number from where calling (in case of disconnecting)
	*3. Telephone number of where the patient can be reached (has to be in note)
	^+^4. Name of Nurse
Patient characteristics	*1. Patient name (full first and last)
	^+^2. Date of birth (month, day and year)
	^+^3. Gender
	4. Past medical history (any documentation of past or ongoing diseases or conditions, this would be chronic conditions such as diabetes, hypertension, sleep apnea)
	5. Allergies (only if in note)
	6. Current medications (any mention of ongoing medications or those already taken for the specific symptom)
Contact characteristics	*1. Caller is clearly identifiable (this may be implied)
	*2. Contact person’s name (caller’s first and last name if not patient)
	*3. Relationship to patient if patient is not caller (must have specific relationship documented)
Reason for call	1. Reason for the encounter
	2. Chief symptoms, complaint, or information desired
	3. Presence or absence of symptoms
	*4. Whether the patient has called before with a similar complaint or information request (recent nurse line call or provider contact for the same complaint within the past 1 week)
Nursing actions	*1. Assessment of symptoms and situation (main symptom stated explicitly)
	^+^2. Specific decision support tool used (clear documentation of any ancillary sources of information used to make decision- including provider input)
	^+^3. Plan of action (clear documentation of disposition, must contain time frame)
	*4. Intervention or information given (any care points, home cares, treatments or protocols documented in note; this doesn’t include advice for what to do with change in symptoms)
	5. Referrals to services, providers (other than the specific disposition- examples would be home health care, specialty appointment, infusion therapy center, etc.)
	6. Coordination of care arranged (conversation/communication with provider, documentation of arrangement for further care such as transferring to appointment coordinator or calling ED- this must be documented, not implied)
Post-triage disposition actions	*1. Patient understanding (documentation that patient or caller understands plan of care)
	*2. Documentation of patient agree or disagree (refusal or agreement with care)
	*3. Nurse's rebuttal documented
	*4. Patient or caller response to rebuttal documented

^+^CDS generated; *CDS prompted.

### Statistical analysis

AAACN dichotomous documentation criteria for each record (Table 2) were adjudicated by majority rule (agreement of 2 of the 3 independent nurse reviewers). The mean score for each dimension was the sum of attained criteria in each dimension divided by the cohort size (50). For the other dichotomous quality measures (Table 3), we were more stringent and required complete (3 of 3) reviewer agreement. Continuous variables (Table 2 note content counts of symptoms, care points and warning signs) had means based on the combined counts of the 3 nurse reviewers.

**Table 2:** Comparison of triage document cohorts by American Academy of Ambulatory Care Nursing (AAACN) triage documentation criteria and triage document content

**Triage content measures**	**Cohort means (SD, median) CDS = clinical decision support**			**Cohort differences (p value) [CI 95%]**		
	**CDS (N = 50)**	**PreCDS (N = 50)**	**No CDS (N = 50)**	**CDS – PreCDS**	**CDS -no CDS**	**No CDS- PreCDS**
**Modified AAACN criteria**						
Encounter characteristics (of maximum 4)	2.88 (0.32, 3)	2.16 (0.37, 2)	2.10 (0.3, 2)	0.72 (<.0001) [0.58 to 0.86]	0.78 (<.0001) [0.65 to 0.91]	−0.06 (0.38) [−0.2 to 0.07]
Patient characteristics (of maximum 6)	3.74 (0.69, 4)	3.92 (0.80, 4)	3.58 (0.73, 3)	−0.18 (0.23) [−0.48 to 0.12]	0.16 (0.26) [−0.12 to 0.44]	−0.34 (0.03) [−.65 to −0.04]
Contact characteristics (of maximum 3)	1.86 (0.99, 1)	1.5 (0.54, 1)	1.14 (0.45, 1)	0.36 (0.03) [0.04 to 0.68]	0.72 (<.0001) [0.41 to 1.0]	−0.36 (0.0005) [−.56 to −0.16]
Reason for call (of maximum 4)	3.08 (0.27, 3)	3.0 (0.29, 3)	2.96 (0.57, 3)	0.08 (0.16) [−0.03 to 0.29]	0.12 (0.184) [−0.05 to 0.30]	−0.04 (0.66) [−0.48 to 0.36]
Nursing actions (of maximum 6)	3.9 (0.58, 4)	2.58 (1.0, 3)	2.52 (1.1, 2)	1.32 (<.0001) [0.99 to 1.65]	1.38 (<.0001) [1.0 to 1.7]	−0.06 (0.78) [−0.48 to 0.36]
Post triage disposition (of maximum 4)	2.0 (0, 2)	1.38 (0.70, 1.5)	0.88 (0.82, 1)	0.62 (<.0001) [0.42 to 0.82]	1.12 (<.0001) [0.89 to 1.4]	−0.5 (0.0015) [−0.8 to −0.20]
**Triage note content**						
Symptom items documented	36.7 (14.3, 36)	10.7 (5.1, 10)	10.2 (6.9, 9. 8)	26.0 (<.0001) [21.8 to 31.4]	26.5 (<.0001) [22.1 to 31.0]	−0.5 (0.68) [ −2.9 to 1.9]
Care points documented	2.79 (2.37, 3)	0.26 (0.7, 0)	0.44 (1.58, 0)	2.53 (<.0001) [1.8 to 3.2]	2.35 (<.0001) [1.6 to 3.2]	0.24 (0.46) [−0.3 to 0.7]
Warning signs documented	1.29 (2.3, 0.33)	0.2 (0.9, 0)	0.01 (0.07, 0)	1.08 (0.0029) [0.8 to 1.4]	1.27 (0.0003) [0.6 to 1.9]	−0.19 (0.16) [−0.5 to 0.08]

**Table 3:** Comparison of triage note cohorts by quality measures

**Note quality measures**	**Cohorts (N = 50)**			**Percent difference between cohorts**		
	**CDS n (%)**	**Pre CDS n (%)**	**No CDS n (%)**	**Pre CDS – CDS (p value)**	**No CDS –CDS (p value)**	**Pre CDS - No CDS (p value)**
**Triage urgency not specific**	0 (0)	17 (34)	21 (42)	34% (<.0001)	42% (<.0001)	−8% (0.54)
**Two or more different triage dispositions**	0 (0)	1 (2)	0 (0)	2% (0.99)	0% (0.99)	2% (0.99)
**Critical symptom indicator missing**	0 (0)	11 (22)	18 (36)	22% (0.0006)	36% (<.0001)	−14% (0.13)
**Major documentation defect**	0 (0)	16 (32)	21 (42)	32% (<.0001)	42% (<.0001)	−10% (0.31)

For continuous variables, we used the t-test for comparisons of the CDS cohort with the controls. The Fisher exact test was used to compare differences in categorical variables from the CDS cohort to the controls.

Analysis was performed between the three cohorts as a pre-post intervention pair (pre-CDS versus CDS), a concurrent intervention versus no intervention pair (CDS versus no-CDS) and a no CDS intervention but training difference pair (no-CDS versus pre-CDS).

## Results

Table 4 shows the triage document cohorts were well matched for gender and triage recommendations. There were no significant differences in sex among the cohorts, and the frequency of ED/911 visit recommendations and appointments recommended were statistically similar. The triage documents of the concurrent control (trained but no CDS) were statistically different in patient age from both the CDS intervention cohort and the pre-CDS cohort.

**Table 4:** Comparison of triage note cohorts by patient demographics and triage note recommendations

**Patient and note disposition characteristics**	**Triage note cohorts (CDS = clinical decision support)**			**Differences between cohorts (p-value, H**_**o**_**: cohorts equal)**		
	**CDS (N = 50)**	**Pre CDS (N = 50)**	**No CDS (N = 50)**	**CDS-Pre CDS**	**CDS- No CDS**	**Pre CDS- NoCDS**
**Mean patient age (SD)**	35.5 (30)	32.9 (28)	53.4 (25)	2.6 (0.65)	17.9 (0.0015)	20.5 (0.0002)
**Female% (n)**	60% (30)	64% (32)	58% (29)	4% (0.84)	2% (0.99)	6% (0.68)
**Recommended emergency/911 disposition% (n)**	8% (4)	10% (5)	0% (0)	2% (0.99)	8% (0.12)	10% (0.056)
**Recommended office appointment disposition% (n)**	50% (25)	48% (24)	36% (18)	2% (0.99)	14% (0.23)	12% (0.31)

The nurses who authored the triage notes were well matched. The nursing group that served as its own control (both pre and post CDS notes) had mean years of employment at Mayo of 21.3 (SD 8.6, median 22.5, range 5.7 to 34.2). The nursing group that served as a concurrent control (CDS trained but no CDS used for the note cohort) had mean years of employment at Mayo of 17.5 (SD 9.1, median 15.6, range 3.2 to 31.6). A parametric test for mean (t-test) and non-parametric test (Wilcoxon) did not show the nurse groups to be significantly different in years of employment at Mayo (p values of 0.07 and 0.14 respectively). Nurses with bachelor’s degrees were the most frequent in both nurse groups, with 19 of 25 (76%) in the pre-post group and 16 of 25 (64%) in the no CDS group. The difference in nurse education level was not significant (p = 0.54).

Table 2 shows the differences in triage documentation when measured by AAACN documentation criteria and by triage document content. Of the 6 categories of triage documentation criteria on the AAACN documentation checklist, 3 of them showed significant improvements with CDS. Triage documentation of patient encounter, nursing actions and post-triage disposition actions were all improved in the CDS documents by significant percents (33%, 51%, and 45%, respectively compared to pre-CDS documentation). Triage documentation was likewise improved when compared to the extra triage training control group (no CDS). The extra training associated with CDS did not improve the documentation and was even associated with a decrease in the documentation quality in patient characteristics, contact characteristics, and post-triage disposition.

There was a significant difference in measures of triage documentation content with CDS (Table 2). When compared to pre-CDS documentation, symptom items, care points, and warning signs all significantly increased in the triage documentation by large amounts (244%, 974%, and 543%, respectively). There were no differences in triage content that we could associate with the extra training involved with CDS.

Table 3 shows the differences in other quality measures between the cohorts. As noted in Methods, all nurse abstractors (3 of 3) had to independently and unanimously agree that there were quality concerns present for the measures in Table 3. Although double disposition endpoints (two different triage dispositions) were rarely observed and were not different between the cohorts, there were other major quality issues that were significantly more frequent in the cohorts not using CDS. In particular there were major documentation defects present in 32% and 42% of the control cohorts and critical symptom indicators were missing in 22% and 36% of the control groups. Major documentation defects were almost exclusively inadequate documentation of symptom assessments.

## Discussion

There were significant improvements in triage documentation attained with nurses using clinical decision support. The pre-CDS and post-CDS triage documentation comparison showed that the same nurses using CDS had marked improvement in their triage documentation. Triage documents authored by nurses trained in CDS but not using it showed no significant improvement. Thus, the improvement in triage documentation appears attributable to CDS and not to a training effect.

Several dimensions of triage documentation improved with CDS. The AAACN standard of triage documentation in the dimensions of patient encounter, nursing action, and post-triage disposition action were all significantly improved. Triage documents produced with CDS also had more documentation of symptom features and so had better documentation of how the nurse arrived at the disposition. Documentation of advice about self-care and warning signs was also significantly greater with CDS. Triage documentation defects were significantly lower with CDS. To our knowledge, this is the first study to quantify these differences in telephone triage documentation attributable to CDS.

From a risk management standpoint, triage documentation is extremely important. Documentation errors were present in 88% of malpractice claims concerning telephone triage [16]. In 84% of the triage malpractice claims there were also faulty triage decisions [16]. Our study shows that CDS in triage would likely decrease the malpractice risk if a claim were filed in a case using CDS. Not only were there significantly more AAACN documentation elements present in the CDS triage records, the CDS documentation had more symptom features listed that could justify the disposition. Using CDS, there were also fewer omissions of critical symptom indicators in the triage documents.

Actors simulating real calls and using scripted symptom scenarios showed that triagists frequently do not ask important symptom indicator questions [10,11]. Our results without CDS are consistent with a similar failure to ask or document important questions. CDS dramatically improved our triage notes. The software prompted the triagist to ask a complete set of symptom indicators for a given symptom and with this CDS prompting, important questions were not forgotten or overlooked.

Triage documentation is likely to increase in importance now that patient portals allow patients to readily view their clinical notes online. Registered patients of Mayo Clinic are currently able to read their triage notes and other clinical notes via the patient portal. At Mayo Clinic, triage documentation is visible on the patient portal in real time (as soon as the triage nurse completes the note). We anticipate that patients will access triage documents as they do clinical notes [17] and use them to review recommendations, warning signs and self-care instructions. CDS increased the documentation of warning signs and self-care points, and patients will be able to benefit from the extra information well after the call is over. We also know that after the triage call, patients only have 60 to 80% adherence to triage recommendations and many are confused about the disposition urgency [18-21]. With the ability to read the triage documentation online, clear dispositions, warning signs and self-care instructions are all present for the patient to review and may improve adherence.

More complete triage documentation is also likely to improve provider efficiency for additional patient evaluation. If a face-to-face visit is required after a triage call, the provider will already have a CDS-generated note that includes an average of 36 positive and negative symptoms methodically acquired with CDS. The provider can use this history to focus more on new information or changes to symptoms rather than repeat an interview already obtained during the triage process. In addition, the more completely documented history obtained with CDS should result in a better documented symptom baseline for longitudinal comparison at a later face-to-face office or ED visit.

Although more documentation is usually good from a risk management standpoint, a potential problem is that it may also be information overload for clinicians who just want a call summary. Documentation of lists of positive and negative symptoms does not necessarily help the readability of the triage note and time-pressed clinicians might dismiss the note altogether. Future study will need to examine how note readability could be improved and how software could highlight the most important positive and negative findings as drivers of triage urgency.

### Strengths and limitations

This study had several strengths. Our study design used a control for nurse training so that we could make stronger conclusions about CDS as the cause for the improvements. We also used standards drawn up by the AAACN, the US nursing professional organization for standards in telephone triage quality and safety. We had a rigorous review process: the triage documentation review had 3 nurses working independently, and majority agreement was required. For the additional quality measures (Table 3) we were even more stringent and required complete agreement from all 3 independent reviewers. The triage document selection process was done sequentially by nurse and so the type or severity of the symptom being addressed was not expected to show any cohort bias. In addition, there were no known epidemics of flu or other illnesses that occurred during April 2008 or May 2010 that would have likely changed the call mix during those times.

A limitation of our study was that our review was restricted to the medical record; we did not review actual telephone calls. A study by Derkx using incognito patients and comparing recorded calls with triage documents found that the triage documentation at times did not match what was heard on the recorded call [22]. Thus, quality of triage documentation does not necessarily reflect quality of triage. A more complete evaluation of differences in triage associated with CDS would require a quality review of recorded calls as has been described by Huibers [23]. In addition to supplying clinical decision support, ExpertRN automatically constructs a triage document that records the symptom assessment questions and responses that the nurse checks off while going through the branching logic. Our design measures mostly documentation support, and nurses who are poor documenters would be helped more by this CDS. From a medical liability standpoint, though, documentation is the number one issue in malpractice claims and this study would be useful for risk managers interested in quantitative evidence of documentation improvement with CDS. Also, for the AAACN documentation standards, the written record is what counts.

A second limitation is that we did not assess the ultimate outcome of these triage decisions. It would be interesting to note if the differences demonstrated in triage documentation quality result in clinically relevant outcome differences. Such a study would logically follow this work.

Triage notes could not be blinded to the CDS intervention. The CDS notes were easily identified by the computer generated syntax and the way the note was organized and worded. Our study design was also retrospective and used real triage notes. We randomly chose notes to review but our samples had some significant differences across the CDS users and the concurrent (no CDS) control.

We were not expecting to observe a significant decrease in documentation criteria from the pre-CDS to the no CDS cohort, but it occurred with criteria for contact characteristics and post-triage disposition documentation (Table 2). The main differences occurred with contact characteristic #3 and post-triage disposition actions #1 and #2 (Table 1). Post-triage disposition criteria #1 and #2 should be at 100%, which they were with the CDS group but were both about 70% pre-CDS and only 44% in the no CDS cohort. These criteria are prompted fields in the CDS that are automatically entered in the note. For the no CDS notes, the training could have conditioned them to be less vigilant about details that would normally be automatically generated by the CDS. The contact characteristics are also prompted fields where the same explanation could hold. Also, the group of nurses in the no CDS group may not have been completely comparable to the pre CDS nurses. The findings could be confounded by the selection process of the no CDS nurses who were not as experienced users of ExpertRN and the experience they did have was variable.

## Conclusion

Telephone triage documentation quality is substantially improved with computerized clinical decision support. CDS applied in triage helps achieve better documentation quality for patient safety and risk management. As more practices allow patients online access to triage notes, it will be important to have well documented notes that patients can refer to for recommendation reinforcement, self-care points and review of warning signs.

## Competing interests

The authors declare that they have no competing interests.

## Authors’ contributions

FN conceived the study and FN, DLC, PV, and RJS contributed to the study design. FN, DDR, KAB and MRL participated in record review, abstraction, and calibration of reviewers. FN did the statistical analysis, was responsible for the study data, and wrote the manuscript. All authors participated in manuscript critical review and revision. All authors read and approved the final manuscript.

## Pre-publication history

The pre-publication history for this paper can be accessed here:

http://www.biomedcentral.com/1472-6947/14/20/prepub

## References

[B1] Osheroff JA Pifer E Teich JM Sittig DF Jenders RA Improving Outcomes with Clinical Decision Support: An Implementer’s Guide 2012 Chicago: Healthcare Information and Management Systems Society

[B2] Conolly P Levine L Amaral DJ Fireman BH Driscoll T TPMG Northern California appointments and advice call center J Med Syst 2005 29 4 325 333 10.1007/s10916-005-5892-z 16178331

[B3] Hildebrandt DE Westfall JM Smith PC After-hours telephone triage affects patient safety J Fam Pract 2003 52 3 222 227 12620177

[B4] Kempe A Bunik M Ellis J Magid D Hegarty T Dickinson LM Steiner JF How safe is triage by an after-hours telephone call center? Pediatrics 2006 118 2 457 463 10.1542/peds.2005-3073 16882795

[B5] Grol R Giesen P van Uden C After-hours care in the United Kingdom, Denmark, and the Netherlands: new models Health Aff 2006 25 6 1733 1737 10.1377/hlthaff.25.6.1733 17102200

[B6] NHS Direct Business Plan NHS Direct Business Plan 2012/13 update http://www.nhsdirect.nhs.uk/About/WhatIsNHSDirect. Last accessed March 20, 2014

[B7] Poole S Providing Telephone Triage and Advice in a Family Practice 2004 Elk Grove Village, Illinois: American Academy of Pediatrics

[B8] Poole SR Creating an after-hours telephone triage system for office practice Pediatr Ann 2001 30 5 268 273 11383466 10.3928/0090-4481-20010501-09

[B9] Richards DA Meakins J Tawfik J Godfrey L Dutton E Heywood P Quality monitoring of nurse telephone triage: pilot study J Adv Nurs 2004 47 5 551 560 10.1111/j.1365-2648.2004.03132.x 15312118

[B10] Derkx HP Rethans J-JE Muijtjens AM Maiburg BH Winkens R van Rooij HG Knottnerus JA Quality of clinical aspects of call handling at Dutch out of hours centres: cross sectional national study BMJ 2008 337 a1264 10.1136/bmj.a1264 18790814 PMC2769520

[B11] Yanovski SZ Yanovski JA Malley JD Brown RL Balaban DJ Telephone triage by primary care physicians Pediatrics 1992 89 4 Pt 2 701 706 1557265

[B12] O’Cathain A Sampson FC Munro JF Thomas KJ Nicholl JP Nurses’ views of using computerized decision support software in NHS Direct J Adv Nurs 2004 45 3 280 286 10.1046/j.1365-2648.2003.02894.x 14720245

[B13] Espensen M Telehealth Nursing Practice Essentials 2009 Pitman, NJ: Anthony J. Jannetti, Inc

[B14] North F Ward WJ Varkey P Tulledge-Scheitel SM Should you search the internet for information about your acute symptom? Telemed J E Health 2012 18 3 213 218 10.1089/tmj.2011.0127 22364307

[B15] Harris PA Taylor R Thielke R Payne J Gonzalez N Conde JG Research electronic data capture (REDCap)–A metadata-driven methodology and workflow process for providing translational research informatics support J Biomed Inform 2009 42 2 377 381 10.1016/j.jbi.2008.08.010 18929686 PMC2700030

[B16] Katz H Kaltsounis D Halloran L Mondor M Patient safety and telephone medicine J Gen Intern Med 2008 23 5 517 522 10.1007/s11606-007-0491-y 18228110 PMC2324141

[B17] Delbanco T Walker J Bell SK Darer JD Elmore JG Farag N Feldman HJ Mejilla R Ngo L Ralston JD Ross SE Trivedi N Vodicka E Leveille SG Inviting patients to read their doctors’ notes: A Quasi-experimental study and a look ahead Ann Intern Med 2012 157 7 461 470 10.7326/0003-4819-157-7-201210020-00002 23027317 PMC3908866

[B18] Bogdan GM Green JL Swanson D Gabow P Dart RC Evaluating patient compliance with nurse advice line recommendations and the impact on healthcare costs Am J Manag Care 2004 10 8 534 542 15352529

[B19] Kempe A Luberti AA Hertz AR Sherman HB Amin D Dempsey C Chandramouli V MacKenzie T Hegarty TW Delivery of pediatric after-hours care by call centers: a multicenter study of parental perceptions and compliance Pediatrics 2001 108 6 E111 10.1542/peds.108.6.e111 11731638

[B20] Moore JD Saywell RM Thakker N Jones TA An analysis of patient compliance with nurse recommendations from an after-hours call center Am J Manag Care 2002 8 4 343 351 11950129

[B21] O’Connell JM Towles W Yin M Malakar CL Patient decision making: use of and adherence to telephone-based nurse triage recommendations Med Decis Making 2002 22 4 309 317 10.1177/0272989X0202200409 12150596

[B22] Derkx H Rethans J-J Muijtjens A Maiburg B Winkens R van Rooij H Knottnerus A ‘Quod scripsi, scripsi.’ The quality of the report of telephone consultations at Dutch out-of-hours centres Qual Health Care 2010 19 6 e1 10.1136/qshc.2008.027920 20584701

[B23] Huibers L Keizer E Giesen P Grol R Wensing M Nurse telephone triage: good quality associated with appropriate decisions Fam Pract 2012 29 5 547 552 10.1093/fampra/cms005 22327415

